# Ultrasound detected increase in optic disk height to identify elevated intracranial pressure: a systematic review

**DOI:** 10.1186/s13089-023-00324-7

**Published:** 2023-05-25

**Authors:** Ghadi Ghanem, David Haase, Agatha Brzezinski, Rikke Ogawa, Parsa Asachi, Alan Chiem

**Affiliations:** 1 David Geffen School of Medicine, University of California, Los Angeles, USA; 2 Department of Emergency Medicine, David Geffen School of Medicine UCLA, Olive View UCLA Medical Center, Los Angeles, USA; 3 UCI Libraries, University of California, Irvine, USA

**Keywords:** Optic disc elevation, Papilledema, Intracranial pressure, ODE, Optic disc, Point of care ultrasound, Ocular ultrasound, Elevated intracranial pressure, Pseudopapilledema, Ocular ultrasound

## Abstract

**Background:**

Elevated intracranial pressure (eICP) is a serious medical emergency that requires prompt identification and monitoring. The current gold standards of eICP detection require patient transportation, radiation, and can be invasive. Ocular ultrasound has emerged as a rapid, non-invasive, bedside tool to measure correlates of eICP. This systematic review seeks to explore the utility of ultrasound detected optic disc elevation (ODE) as an ultrasonographic finding of eICP and to study its sensitivity and specificity as a marker of eICP.

**Methods:**

This systematic review followed the preferred reporting items for systematic reviews and meta-analyses guidelines. We systematically searched PubMed, EMBASE, and Cochrane Central for English articles published before April 2023; yielding 1,919 total citations. After eliminating duplicates, and screening the records, we identified 29 articles that addressed ultrasonographically detected ODE.

**Results:**

The 29 articles included a total of 1249 adult and pediatric participants. In patients with papilledema, the mean ODE ranged between 0.6 mm and 1.2 mm. Proposed cutoff values for ODE ranged between 0.3 mm and 1 mm. The majority of studies reported a sensitivity between 70 and 90%, and specificity ranged from 69 to 100%, with a majority of studies reporting a specificity of 100%.

**Conclusions:**

ODE and ultrasonographic characteristics of the optic disc may aid in differentiating papilledema from other conditions. Further research on ODE elevation and its correlation with other ultrasonographic signs is warranted as a means to increase the diagnostic accuracy of ultrasound in the setting of eICP.

**Supplementary Information:**

The online version contains supplementary material available at 10.1186/s13089-023-00324-7.

## Introduction

Elevated intracranial pressure (eICP) is an emergent condition that requires prompt identification and management. eICP may present as a result of many etiologies including mass lesions, traumatic bleeds, hydrocephalus, obstruction, brain oedema, or in some cases, idiopathically [1]. eICP is difficult to detect, and patients may present with vague symptoms of headache, nausea and vomiting, visual disturbances, or decreased level of consciousness [2]. These conditions often result in a decrease of cerebral perfusion as a consequence of the eICP—thereby contributing to cerebral ischemia [3]. Identification of this perfusion–demand mismatch can allow for the initiation of therapies to decrease hypercarbia, prevent hypotension, and control secondary seizures [3]. In patients with traumatic brain injury, early detection and monitoring of eICP is of upmost importance [4–6].

The gold standard of eICP detection and monitoring has historically been quite invasive—requiring extra-ventricular drainage or intra-ventricular catheters. This began to be integrated into practice two decades ago, when a report detailing the benefits of ICP monitoring was published by The American Association of Neurological Surgeons [7, 8]. Often, however, the invasive nature of these tests discourages their utilization. In lieu, edema of the optic disc has long been used as a proxy of ICP—since the meninges are continuous with the optic nerve sheath. Fundoscopy, in the hands of an experienced provider, may be a useful tool in detecting swelling of the optic disc—however, it is difficult to discriminate between true papilledema, pseudopapilledema, and other ocular conditions as their fundoscopic presentations often overlap [9].

**Fig. 1:**
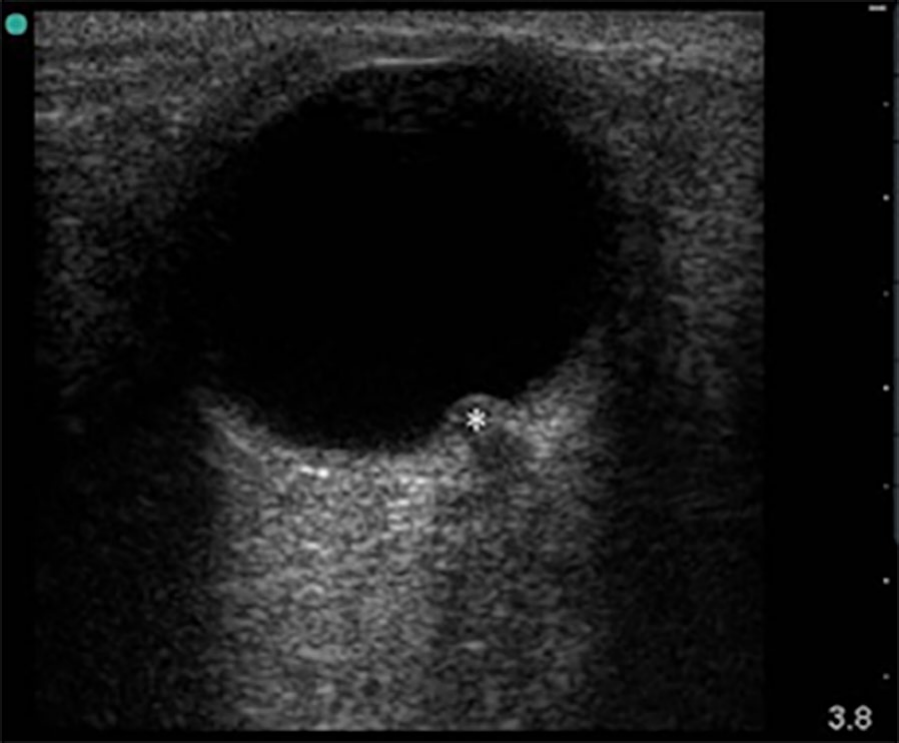
Elevation of the optic disc, marked with a star on B scan ocular ultrasound

Increasing ICP transmits across the subarachnoid space and causes accumulation of cerebrospinal fluid in the anterior part of the optic nerve, thereby causing distention and eventually papilloedema [10, 11]. This expansion of fluid can be detected by B scan ultrasound as widening of the optic nerve sheath diameter (ONSD), and/or potentially as optic disc elevation (ODE). On ultrasound, ODE is a contoured, hyperechoic prominence into the vitreous (Fig. 1). The height of the prominence has been found to be correlated with the severity of edema [12]. Ultrasound can be performed rapidly at the bedside and in emergent situations—without delaying treatment or exposing the patient to harmful radiation.

Another important condition which can present with elevation of the optic disc is pseudopapilledema or optic disc edema that is not associated with eICP or swelling of the nerve. Pseudopapilledema can be divided into categories such as structural pseudopapilledema or optic disc drusen (ODD), the latter of which is more common with an estimated prevalence of 2% in the general population [13]. ODD is the accumulation of vitreous material deposits on the optic disc. ODD is often initially asymptomatic and embedded in the optic disc, but can progress to cause swelling and calcification [14]. This can be detected on B-scan ultrasound as an ovoid hyperechogenic lesion at the junction of the retina and the optic nerve [15, 16].

One of the more heavily studied means of discriminating between papilledema and pseudopapilledema is by measuring the optic nerve sheath. Measurement of the ONSD 3 mm behind the retina has yielded a pooled sensitivity of 0.90, a pooled specificity of 0.85, and a pooled diagnostic odds ratio of 51 in a 2011 meta-analysis by Dubourg et al. [17]. It has therefore gained large popularity as both a diagnostic and monitoring modality for eICP. However, a few studies in unique patient populations have suggested that there was insufficient or moderate correlation between ONSD and eICP [18, 19]. Other literature has identified that although the method has very high sensitivity, the specificity may be limited at 75% [20].

We thus sought to explore the utility of ultrasound detected ODE as another ultrasonographic finding of eICP—and to study its sensitivity and specificity as an independent marker of eICP, or in conjunction with ONSD.

## Methods

PICO question: In all patients presenting to the hospital, how effective is ultrasound, as compared to more invasive methods, at identifying papilledema or pseudopapilledema.

### Approach

We conducted this systematic review in accordance with the Preferred Reporting Items for Systematic Reviews and Meta-Analysis (PRISMA) guidelines [21, 22] to identify articles that utilize ultrasound to determine optic disc elevation and its correlation with papilledema or pseudopapilledema. Inclusion and exclusion criteria were determined in advance and documented in a protocol. The review was registered (CRD 42022251595) in the International Prospective Register of Systematic Reviews [PROSPERO] on 03 December, 2021.

### Literature search strategy

**Fig. 2:**
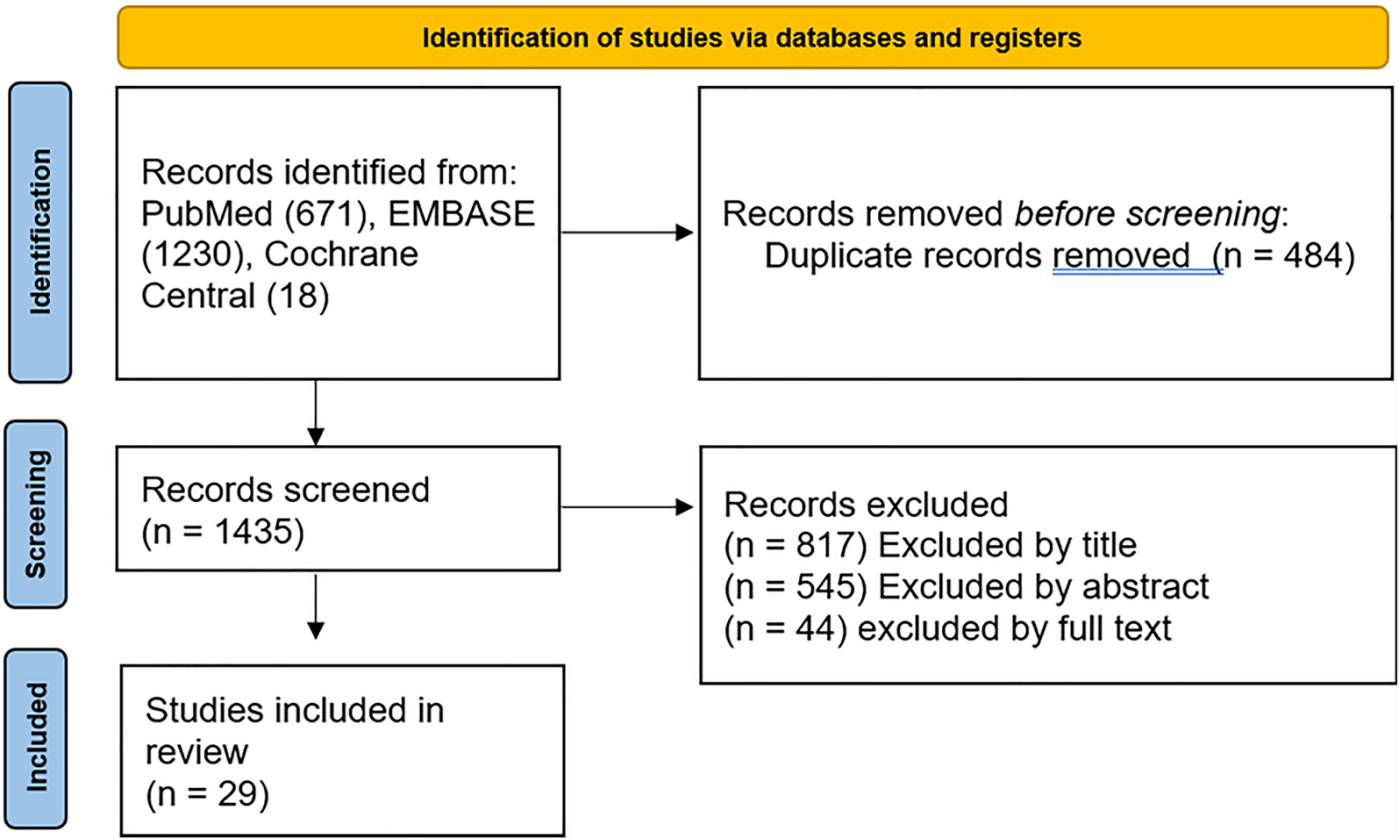
Flowchart of study selection for systematic review

We systematically searched the following databases for relevant publications: PubMed, EMBASE (Embase.com), and Cochrane Central (Cochranelibrary.com) on 7 April 2021. We included all articles which met inclusion criteria and were published from inception of the online database to this date. The search strategy used a combination of keywords and subject headings for ultrasound, optic nerve, and edema, with limits to English language and human subjects (see Appendix section). Across all three databases 1703 total citations were found in the initial search. After deduplication, total citations retrieved were reduced to 1267 (Fig. 2).

### Literature review update

Prior to publication, a second literature review was conducted using the same search strategy and databases. The search was conducted on April 10, 2023. All articles published between April 8, 2021 and December 31, 2023 were included, yielding 216 total articles across all three databases. Duplicates were filtered, reducing total articles to 175.

### Inclusion and exclusion criteria

We included all English articles published before April 10, 2023 that correlated optic disc height to papilledema or pseudopapilledema in human subjects. Case reports of only one patient were excluded. We did not restrict our search to any dates, and all genders, ages, and locations were included.

### Data collection and data items

We created a data extraction sheet based on the Cochrane Consumers and Communication Review Group’s data extraction template [24]. Researchers extracted the predetermined information from each study that had been selected for inclusion. One reviewer conducted primary extraction of relevant data from each study. A second reviewer independently confirmed the accuracy of extracted data. We collected participant demographics (age, gender, ethnicity), sample size, study setting, inclusion and exclusions criteria, ultrasound information (equipment/scanning technique/practitioner conducting the scan), the study’s gold standard or comparison point, temporal information (when and how often patients are being scanned), the study’s definition for optic disk elevation, pathology or disease being studied, study outcomes, risk of bias (randomization, blinding, selective reporting) as well as any raw data published by the authors. No efforts were made to make any assumptions about any missing information.

### Outcomes

The outcome of primary interest was the correlation between optic disc height and measures of eICP. Secondary outcomes included the qualitative assessment of ultrasonographic presentations of papilledema or pseudopapilledema.

### Quality assessment

The quality of the included studies as well as any risk of bias were assessed using the Quality Assessment Of Diagnostic Accuracy Studies-2 (QADAS-2) [25].

### Planned methods of analysis

All analyses were conducted on the native form of the data extracted. When necessary, sensitivity, specificity and confidence intervals were calculated from the raw data or the study’s results. All analyses were reported qualitatively based on the data presented in each article. Figures were created using Microsoft Corporation, 2018. (Microsoft Excel, Available at: https://office.microsoft.com/excel) or MedCalc for Windows, version 19.4 (MedCalc Software, Ostend, Belgium). Meta analysis was not planned due to the heterogeneity of collected data 1).

## Results

### Study selection

In the initial search, PubMed yielded 542 articles, EMBASE yielded 1146, and Cochrane Central yielded 15. Articles were imported into Mendeley [23], a citation manager, and duplicates were automatically removed—yielding 1260 unique articles. Upon updating the literature search in 2023, PubMed yielded an additional 129 articles, Embase yielded an additional 84, and Cochrane an additional 3. Duplicates were removed resulting in 175 unique titles. In the first phase, two reviewers (GG, DH or PA) independently screened the articles by title to ensure that the articles were relevant to the inclusion criteria. The reviewers were blinded from each other’s decisions, and in the event of a disagreement, an ultrasound fellowship trained attending physician (AC) acted as the tie-breaking vote. Deviations were found to be minimal. The articles were re-screened by abstract, then by full-text using the same procedure. (GG, DH,AB, and PA).

### Study characteristics

A total of twenty nine studies published between 1994 and 2023 were selected for inclusion in this systematic review [12, 14, 26–52] (Table 1). Six out of the twenty nine studies were case series [28, 30, 37, 43, 45, 46]. Furthermore, seven studies were conducted in pediatric patients only [27, 28, 30, 31, 37, 48, 49], two had a mixed population of children and adults [14, 42], and the remainder were conducted in adult patients only. All studies utilized B scan ultrasonography with high-frequency transducers, ranging in frequency from 3 MHz (megahertz) to 20 MHz. One study also conducted A scan ultrasonographic measurements [42].

**Table 1:** List of studies included in systematic review

Title	Author	Participants
A Prospective Study of Optic Nerve Ultrasound for the Detection of Elevated Intracranial Pressure in Severe Traumatic Brain Injury	Agrawal et al. [26]	120 adults
Accuracy of Diagnostic Imaging Modalities for Classifying Pediatric Eyes as Papilledema Versus Pseudopapilledema	Chang et al. [27]	19 children
Diagnostic Value of Systematic Imaging Examination in Embedded Optic Disc Drusen in Adolescents with Mild Visual Impairment	Jia et al. [14]	11 (13–23 y.o.)
Emergency point-of-care ultrasound detection of papilledema in the pediatric emergency department	Ben-Yakov et al. [28]	4 children
Feasibility and usefulness of ultrasonography in idiopathic intracranial hypertension or secondary intracranial hypertension	Lochner et al. [29]	42 adults
Four-dimensional ultrasound imaging in neuro-ophthalmology	Titianova et al. [12]	30 adults
Identification of optic disc elevation and the crescent sign using point-of-care ocular ultrasound in children	Marchese et al. [30]	4 children
Identification of Optic Nerve Swelling Using Point-of-Care Ocular Ultrasound in Children	Marchese et al. [31]	76 children
Low energy diet and intracranial pressure in women with idiopathic intracranial hypertension: prospective cohort study	Sinclair et al. [32]	25 adult women
Ocular ultrasonography for diagnosing increased intracranial pressure in patients with severe preeclampsia	Simenc et al. [33]	60 adult women
Ocular ultrasound for monitoring pseudotumor cerebri syndrome	Lochner et al. [34]	22 adults
Optic nerve sheath enlargement in acute intracranial hypertension	Hansen et al. [35]	36 adults
Point-of-care ocular ultrasound to detect optic disc swelling	Teismann et al. [36]	14 adults
Point-of-care ultrasonography for the identification of 2 children with optic disc drusen mimicking papilledema	Braun et al. [37]	2 children
Role of Orbital Ultrasound in the Assessment of Clinically Detected Papilledema	Mohson et al. [38]	80 adults
Sonographic assessment of optic nerve and ophthalmic vessels in patients with idiopathic intracranial hypertension	Ebraheim et al. [39]	54 adults
Sonographic assessment of the optic nerve and the central retinal artery in idiopathic intracranial hypertension	Jeub et al. [40]	39 adults
Sonographic assessment of the optic nerve sheath in idiopathic intracranial hypertension	Bäuerle et al. [41]	35 adults
The efficacy of optic nerve ultrasonography for differentiating papilloedema from pseudopapilloedema in eyes with swollen optic discs	Neudorfer et al. [42]	44 adults + children
The usefulness of multimodal imaging for differentiating pseudopapilloedema and true swelling of the optic nerve head: a review and case series	Chiang et al. [43]	5 adults
Ultrasonographic evaluation of optic disc swelling: comparison with CSLO in idiopathic intracranial hypertension	Tamburrelli et al. [44]	36 adults
Ultrasound assessment of optic disc edema in patients with headache	Daulaire et al. [45]	3 adults
Utility of Point-of-Care Ultrasound in the Diagnosis of Idiopathic Intracranial Hypertension in the Emergency Department	Huo et al. [46]	5 adults
Bedside ocular ultrasonography for diagnosing increased intracranial pressure in patients with leptomeningeal metastases from non-small-cell lung cancer	Jiang et al. [47]	137 adults
Pediatric point-of-care ultrasound of optic disc elevation for increased intracranial pressure: A pilot study	Tessaro et al. [48]	40 children
Reliability and feasibility of optic nerve point-of-care ultrasound in pediatric patients with ventricular shunts	Gauthey et al. [49]	81 children
Sonographic and ophthalmic assessment of optic nerve in patients with idiopathic intracranial hypertension: A longitudinal study	Knodel et al. [50]	44 adults
Transorbital sonography: A non-invasive bedside screening tool for detection of pseudotumor cerebri syndrome	Korsbæk et al. [51]	74 adults
Ultrasonic optic disc height combined with the optic nerve sheath diameter as a promising non-invasive marker of elevated intracranial pressure	Yu et al. [52]	107 adults

The included studies had a global distribution with eleven studies having been conducted in Europe, ten in North America, five in Asia, and one each in Oceania and Africa. There was also heterogeneity as to the setting for each study; seven studies were conducted in the emergency department, seven were conducted in the department of neurology, four were conducted in the department of ophthalmology or an eye center, and two were conducted in a neurotrauma or intensive care unit. Other settings included a perinatal center, clinical research facility, a mix of settings, or did not specify where enrollment was conducted.

### Quality analysis

The included studies examining optic nerve sheath diameter and optic disc elevation varied in their reference standard used for evaluating eICP. One study used ICP monitoring as the reference standard [26]. Other studies used elevated opening pressure on Lumbar Puncture (LP) as their reference standard [27, 32, 38, 47, 50–52]. Not all studies were evaluating elevated ICP, as some studies examined patients for optic disc abnormalities [12, 14, 27, 31, 36, 37, 42, 44, 45]. The included studies were a mix of observational studies [14, 26, 27, 29, 36, 42, 49], case control [12, 33, 35, 38–41, 44, 50, 51], cohort studies [29, 31, 32, 47, 48, 52] and case reports [28, 30, 37, 43, 45, 46] with their own inherent limitations. They ranged in sample sizes from a case report of two patients to a study of 137 patients. Most of the studies were on adult patients though seven studies involved only pediatric patients.

The QADAS-2 tool (Additional file 1) identified that in 23 out of the 29 studies, selection of patients may have introduced bias. However, all studies were low risk that the included patients did not match the review question. The conduct or interpretation of the index test had a high risk of introducing bias in nine studies, and unclear in three. All studies were low risk of concern that the index test, its conduct or interpretation differed from the review question. The reference standard had low risk of introducing bias in thirteen studies, while the remainder were high risk or did not use a reference standard. Of the studies which employed a reference study, all of them were low risk that the target condition as defined by the reference standard did not match the review question. Finally, the patient flow had a high risk of introducing bias in nine studies, low risk in thirteen studies, and was unclear in seven.

### Optic disc height

Mean optic disc height in patients with papilledema ranged from 0.6 mm to 1.2 mm, with some studies documenting a slight variation between the Optic Disc Height (ODH) in the left eye and the right eye. (Table 2) One study reported the ODH as a median of 0.95 mm in the right eye and 1.0 mm in the left eye [34]. Yu et al. 2023 reported a median of 0.81 mm [52].

**Table 2:** Reported optic disc heights in patients with papilledema

Author	Reported mean optic disk height (mm)
Lochner et al. [29]	0.8 (SD = 0.43 mm) on the right side 0.8 (SD = 0.38 mm) on the left side
Sinclair et al. [32]	1.02 (SD = 0.3 mm)
Ebraheim et al. [39]	1.1 (SD = 0.3 mm)
Jeub et al. [40]	0.9 (SD = 0.1 mm) on the right side 0.9 (SD = 0.1 mm) on the left side
Bäuerle et al. [41]	1.2 (SD = 0.3 mm) on the right side 1.2 (SD = 0.3 mm) on the left side
Tamburrelli et al. [44]	1.17 (SD = 0.38 mm)
Knodel et al. [50]	0.6 (SD = 0.5 mm) in the right eye 0.7 (SD = 0.4 mm) in the left eye
Korsbæk et al. [51]	0.9 (SD = 0.4 mm)
Lochner et al. [34]	Median: 0.95 mm (0.70–1.43) on the right side Median: 1.00 mm (0.58–1.3) on the left side
Yu et al. [52]	Median: 0.81 mm (0.60–1.06)

### Sensitivity and specificity

**Fig. 3:**
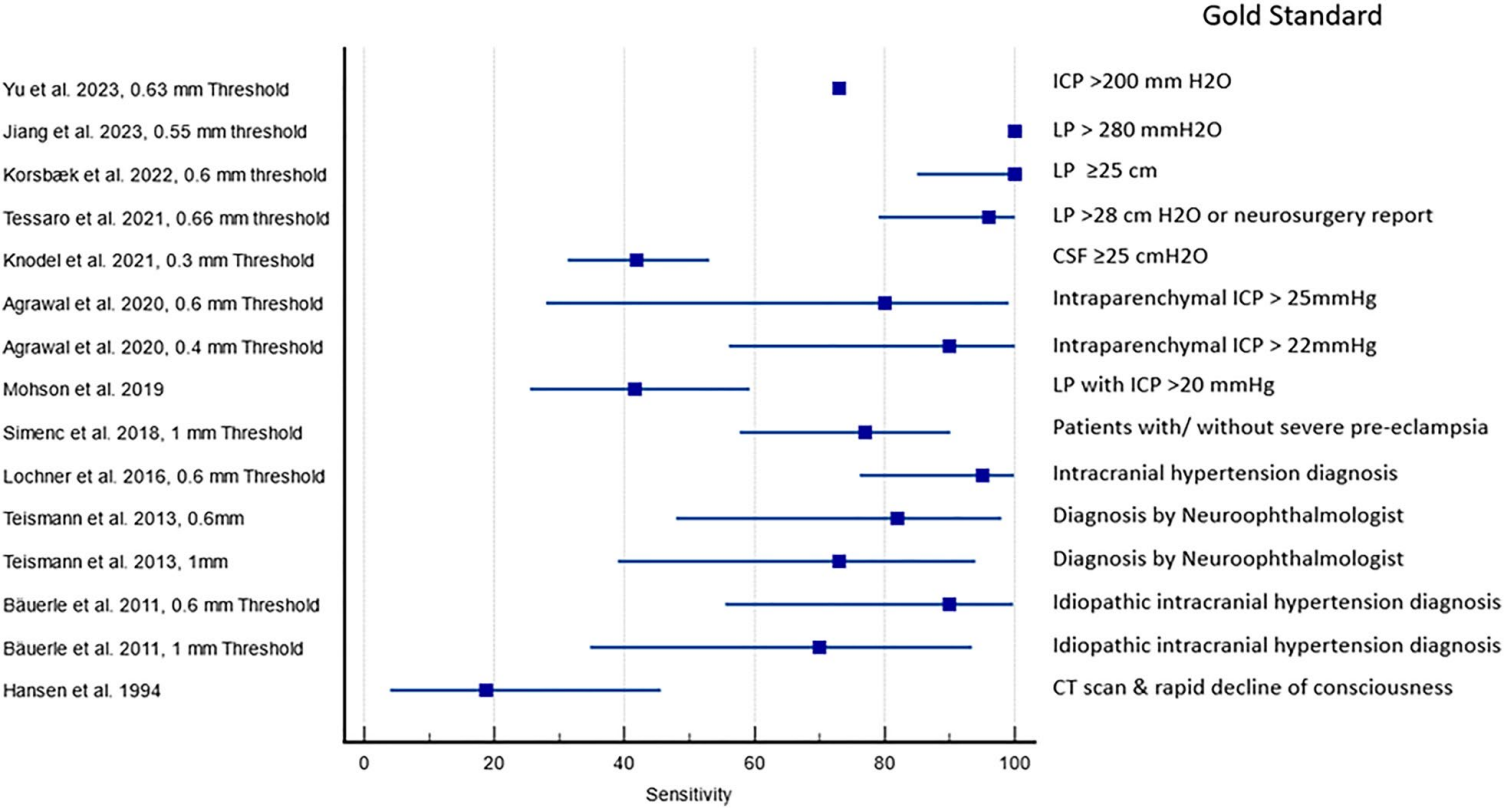
Reported and calculated sensitivity of ultrasound detected optic disc elevation in detecting papilledema

**Fig. 4:**
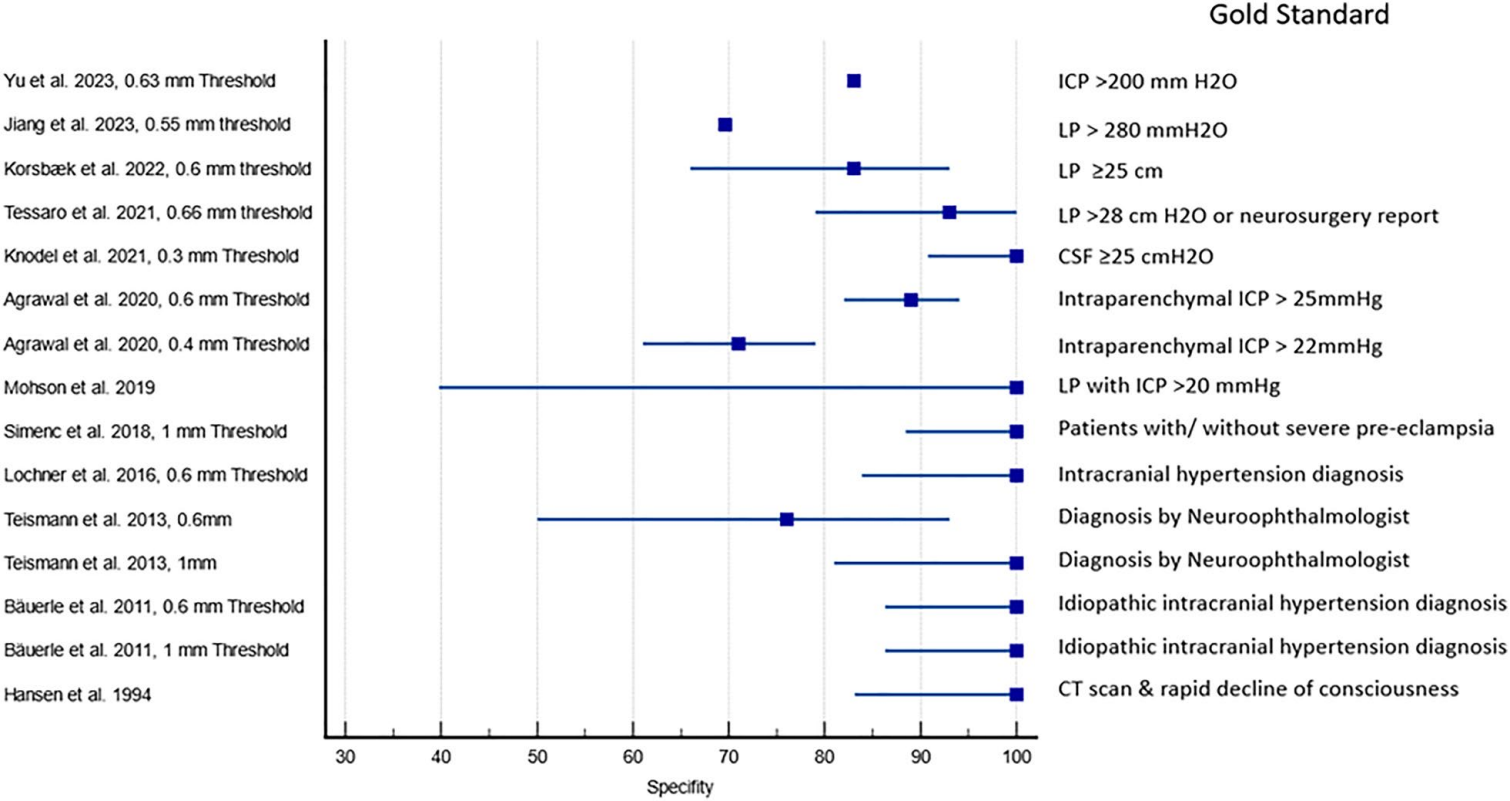
Reported and calculated specificity of ultrasound detected optic disc elevation in detecting papilledema

Figures 3 and 4 show the sensitivity and specificity of ultrasound detected ODE in the identification of papilledema. The gold standards of the studies varied significantly, and included a diagnosis of Idiopathic intracranial hypertension (IIH), papilledema on fundoscopy, elevated Cerebrospinal fluid (CSF) opening pressure during lumbar puncture, evidence of elevated ICP on Computed tomography (CT) scan, amongst other endpoints. Some studies did not explicitly provide sensitivity and specificity values. These values were calculated by our study team based on the provided information or raw data. One study [41] did not provide ODE cut-off values, so the study team assigned values of 0.6 mm and 1 mm arbitrarily for data analysis based on the majority of studies having used these values. The sensitivity and specificity of two studies was extrapolated from reported area under the curve graphs for ODE cut off values. Confidence intervals could not be discerned [47, 52].

### Other papilledema results

Two studies found that ODE and CSF opening pressure had no correlation (Spearman’s *ρ* =  − 0.016, *P* = 0.94) [34] and (*r* = 0.27, *p* = 0.186) [50]. Four studies found a positive correlation between ODE and CSF (*r* = 0.383, *p* = 0.025) [47] (*r* = 0.77, *R*^2^ = 0.59, *p* < 0.001) [51], (*r* = 0.613, *p* < 0.001) [52] and (*r* = 0.572, *p* = 0.004) [39]. In a study by Ebraheim et al. 2018, re-measuring ODE and ONSD at 4 week post-lumbar puncture found a significantly decreased ONSD (6.8 ± 0.5 vs. 6.4 ± 0.6 mm; *p* = 0.006) and ODE (1.1 ± 0.3 vs. 0.9 ± 0.9 mm; *p* = 0.006) [39].

Three studies explored the parameters associated with combining ONSD and ODH measurements for the detection of eICP. Marchese et al. used an ONSD cutoff of 4.5 mm and ODE > 1 mm, yielding a sensitivity of 90% and specificity of 55% [31]. Yu et al. found the sensitivity and specificity of ODH > 0.63 mm combined with the ONSD > 4.68 mm in the diagnosis of elevated ICP were 93% and 92%, respectively [52]. Jiang et al. proposed an ODH > 0.055 cm combined with the ONSD > 0.615 cm yielded a sensitivity of 87.50% and a specificity of 85.70% [47].

In terms of feasibility and accuracy, Marchese et al. demonstrated that optic disc point of care ultrasound was able to visualize the optic disc and allow for swelling evaluation in 100% of cases, whereas direct fundoscopy was only successful in 40% of examinations [31]. Tamburelli et al. established that the Test–retest variability for ODE is consistent with a variability of 0.09 ± 0.04 mm (range, 0.02–0.16 mm) [44]. Gauthey et al. found intraclass correlation coefficient for ODE to be 0.81 (95% CI 0.75–0.89) for right eyes and 0.85 (95% CI 0.79–0.91) for left eyes [49].

### Optic disc drusen/pseudopapilledema

The ultrasonographic presentation of pseudopapilledema can complicate the diagnosis of true papilledema. Chang et al. describes how superficial ODD is visualized as hyperechoic lesions on the optic nerve head, with posterior shadowing. However, buried ODD can present as an elevated optic nerve head, without a hyperechoic mass in almost 70% of patients. This causes B-scan ultrasonographic appearance of papilledema to be indistinguishable from eyes with suspected buried ODD without calcification [27]. Hence, a typical presentation of ODD with irregular flat protuberances with strong discoid echoes is only present in about 55% of patients [14]. Chang et al. reported that the accuracy of ultrasound in distinguishing papilledema from pseudopapilledema is therefore 74%. Papilledema is misinterpreted as pseudopapilledema in 60% of cases, and pseudopapilledema is misinterpreted as papilledema in 12% of cases [27]. The use of ONSD or optic nerve width may have the ability to increase the sensitivity however [41].

### Case reports and information gained

We included 6 case series with a total of 23 patients. Many of these case reports demonstrate the utility of ocular sonography in an emergent setting, especially with a pediatric population in which traditional fundoscopy may be difficult [28, 30]. One case report also details how pseudopapilledema may be identified in this population using ultrasound [37].

## Discussion

In this review, we identified various presentations and underlying pathologies of ultrasonographically detected elevated ODH. In looking at the collected data from the included studies, it is evident that ultrasound detected ODE is a feasible, non-invasive test with high specificity and sensitivity that may aid physicians in monitoring and screening for eICP. The ability to rapidly rule-in papilledema in a patient presenting with vague symptoms may significantly improve outcomes.

Out of the included studies which reported a sensitivity and specificity for ODE, there was wide variation in cutoff values for ODE which were proposed; these ranged from 0.3 mm to 1 mm. In studies that included raw data or evaluated both the 0.6 mm and 1 mm cutoff, opting for the 0.6 mm threshold resulted in a 9–20% increase in sensitivity. This was at an expense of a 0–24% decrease in specificity. Since eICP is an acute condition that must be ruled out on patient presentation, it would benefit from a lower threshold to ensure that no patients are missed. Studies which conducted an area under the curve analysis for optimal sensitivity and specificity of ODH determined cut-off values between 0.55 and 0.66 mm in their respective patient populations. It is thereby evident that more research to determine an optimal cutoff value is warranted. Furthermore, it must be considered that certain population groups, such as pediatric patients or patients with unique chronic conditions resulting in eICP, may require a modification of this cut-off value.

Another means to increase the sensitivity and specificity would be the validation of other ultrasonographic signs of eICP. One such indicator, the crescent sign, shows hypoechoic fluid surrounding the optic nerve in the cross-sectional view (vertical transverse orientation) [30]. One study has identified that the crescent sign had a sensitivity of 92% in a sample of 50 patients with known papilledema [53]. Another such technique is the 30 degree test. This test is performed on patients whose ONSD is suspicious for papilledema. The ONSD is measured once with the patient looking forward, and again with the patient’s gaze fixated 30 degrees from the primary gaze towards the ultrasound probe. A decrease in the ONSD of more than 10% is a positive finding indicative of fluid rather than solid thickening [54]. This test is often used to increase the accuracy of ONSD measurements in diagnosing true papilledema.

The combination of these techniques may yield a highly sensitive and specific means of non-invasively monitoring eICP. A randomized controlled trial of patients with ICP monitoring vs imaging and clinical examination yielded no significant difference in both 14 day and 6 month mortality. Furthermore, the length of stay and distribution of serious adverse events were similar in both groups [55]. Patients with ICP monitoring had a significantly increased risk of pneumonia, renal failure, infections, increased duration of mechanical ventilation and intensive care unit length of stay, and decreased functional outcome [56, 57]. Of further concern is the risk associated with placement of the ICP monitoring device. Notable complications include a risk of hemorrhage [58, 59], bacterial infection [60], and misplacement [61]. Although these risks have been reduced in recent years due to technological advancements, they remain significant enough to warrant further analysis as to how non-invasive eICP monitoring can serve as an appropriate adjunct. Given the data from these studies, ODE may be able to supplement or complement traditional imaging studies, such as the CT scans utilized in the randomized controlled trial by Chestnut et al. [55]. This may be especially useful in communities with limited resources, situations where repeat radiation exposure is contraindicated, or in the rapidly emerging fields of space medicine and space flight.

### Limitations

There are some limitations to this systematic review that should be acknowledged. Due to the invasive nature of lumbar puncture and intraparenchymal measurement, the diagnosis of eICP was not always established by CSF pressure analysis—leading to considerable heterogeneity between the studies. Furthermore, the majority of studies determined their own cutoff values for ODE, and raw data was limited. Due to these factors, we were unable to compare across studies, and were unable to conduct a meta-analysis with the included data. We made attempts to present the data as accurately as possible in order to encourage further research on the subject of ODE in the setting of eICP; however, we could not draw definitive conclusions on optimal cutoffs or pooled sensitivity and specificity.

It is important to note that eICP can take time to cause ODE, and, therefore, may not always be applicable in the acute setting. Patients with acute traumatic brain injury, intracranial hemorrhage, or other acute onset condition may not have developed ODE at the time of their evaluation [36, 62]. Studies are scarce in their description of ODE timeline after an acute event, hence, more research is necessary to further elucidate this point.

Although papilledema and pseudopapilledema are the two most common conditions that may present with ODE, more rare etiologies include lesions and cancerous growths on the optic disc. One of these lesions is melanocytoma, a rare growth of hyperpigmented nevus cells, which can be identified by ophthalmoscopy as a dark lesion on the optic disc [63]. On ultrasound, these appear as optic disc elevations with a mean height between 1.3 mm and 1.99 mm, and a mean diameter between 2.1 mm and 3.1 mm [64–66]. Our study did not include these studies because of their unique ultrasonographic and ophthalmoscopic characteristics that will not usually overlap with papilledema.

## Conclusions

ODE and ultrasonographic characteristics of the optic disc may aid in differentiating papilledema from other ocular conditions. Furthermore, ODE has the potential to be used in conjunction with ONS diameter as a correlate of eICP. Larger, prospective studies are needed to assess the role of ODE elevation and its correlation with other ultrasonographic signs, such as ONS diameter, the crescent sign and the 30-degree test in patients with suspected eICP.

### Supplementary Information

**Additional file: 1.** Bias analysis.

## Data Availability

The data sets used and/or analyzed during the current study are available from the corresponding author on reasonable request.

## Search strategies

Search conducted: April 7, 2021.

Repeat search conducted on: April 10, 2023.

## PubMed

Retrieval: 542 unique citations retrieved.

Update: 129 additional citations.

Strategy:(("ultrasound*"[Title/Abstract] OR "ultrasonograph*"[Title/Abstract] OR "sonograph*"[Title/Abstract] OR "sonogram*"[Title/Abstract] OR "ultrasonic*"[Title/Abstract] OR "Ultrasonography"[MeSH Terms] OR "Ultrasonics"[MeSH Terms]) AND (("Optic disc"[Title/Abstract] OR "Optic Disk"[MeSH Terms] OR "Optic Nerve"[MeSH Terms:noexp] OR "Optic Nerve"[Title/Abstract]) AND ("elevat*"[Title/Abstract] OR "raise*"[Title/Abstract] OR "bulg*"[Title/Abstract] OR "Edema"[Title/Abstract] OR "swelling"[Title/Abstract] OR "Edema"[MeSH Terms] OR "Papilledema"[Title/Abstract] OR "Papilledema"[MeSH Terms] OR "Pseudopapilledema"[Supplementary Concept] OR "Pseudopapilledema"[Title/Abstract] OR "pseudo-papilledema"[Title/Abstract])) AND "English"[Language]) NOT ("animals"[MeSH Terms] NOT "humans"[MeSH Terms]).

## EMBASE

Retrieval: 715 unique citations (1,146 citations retrieved, 431 overlapping with other search results).

Strategy:((ultrasound*:ti,ab OR ultrasonograph*:ti,ab OR sonograph*:ti,ab OR sonogram*:ti,ab OR ultrasonic*:ti,abOR 'echography'/exp OR 'ultrasound'/exp)AND (('optic disk'/exp OR 'optic nerve'/exp OR 'optic disc':ti,ab OR 'optic nerve':ti,ab) AND('edema'/exp OR 'papilledema'/exp OR 'pseudopapilledema'/exp OR elevat*:ti,ab OR raise*:ti,ab OR bulg*:ti,ab OR edema:ti,ab OR swelling:ti,ab OR papilledema:ti,ab OR pseudopapilledema:ti,ab)) AND [english]/lim) NOT ('animal experiment'/exp NOT ('human experiment'/exp OR 'human'/exp)).

## Cochrane CENTRAL

Retrieval: 10 unique citations (15 citations retrieved, 5 overlapping with other search results).

Strategy:((("ultrasound*" OR "ultrasonograph*" OR"sonograph*" OR "sonogram*" OR "ultrasonic*"):ti,ab,kw OR "Ultrasonography"[MeSH Terms] OR "Ultrasonics"[MeSH Terms]) AND ((("Optic disc" OR "Optic Nerve"):ti,ab,kw OR "Optic Disk"[MeSH Terms] OR "Optic Nerve"[MeSH Terms:noexp]) AND (("elevat*" OR "raise*" OR "bulg*" OR "Edema" OR "swelling" OR "Pseudopapilledema" OR "pseudo-papilledema" OR "Papilledema"):ti,ab,kw OR "Papilledema"[MeSH Terms] OR "Edema"[MeSH Terms])).

## Abbreviations

eICP: Elevated intracranial pressure
ICP: Intracranial pressure
ONSD: Optic nerve sheath diameter
ODE: Optic disc elevation
ODD: Optic disc drusen
PRISMA: Preferred Reporting Items for Systematic Reviews and Meta-Analysis
QADAS-2: Quality Assessment Of Diagnostic Accuracy Studies-2
MHz: Megahertz
LP: Lumbar puncture
ODH: Optic Disc Height
IIH: Idiopathic intracranial hypertension
CSF: Cerebrospinal fluid
CT: Computed tomography

## Code availability

Data Code is not publicly available for this study.
